# Biofilm-Related Infections in Gram-Positive Bacteria and the Potential Role of the Long-Acting Agent Dalbavancin

**DOI:** 10.3389/fmicb.2021.749685

**Published:** 2021-10-22

**Authors:** Alessandra Oliva, Stefania Stefani, Mario Venditti, Enea Gino Di Domenico

**Affiliations:** ^1^Department of Public Health and Infectious Diseases, “La Sapienza” University of Rome, Rome, Italy; ^2^Laboratory of Molecular Medical Microbiology and Antimicrobial Resistance Research (Mmarl), Department of Biomedical and Biotechnological Sciences (Biometec), University of Catania, Catania, Italy; ^3^Microbiology and Virology, IRCCS San Gallicano Institute, Rome, Italy

**Keywords:** biofilm, dalbavancin, *Staphylococcus aureus*, Gram-positive, skin, soft tissue infections

## Abstract

Infections caused by Gram-positive bacteria are a major public health problem due to their increasing resistance to antibiotics. *Staphylococcus* and *Enterococcus* species’ resistance and pathogenicity are enhanced by their ability to form biofilm. The biofilm lifestyle represents a significant obstacle to treatment because bacterial cells become highly tolerant to a wide range of antimicrobial compounds normally effective against their planktonic forms. Thus, novel therapeutic strategies targeting biofilms are urgently needed. The lipoglycopeptide dalbavancin is a long-acting agent for treating acute bacterial skin and skin structure infections caused by a broad range of Gram-positive pathogens. Recent studies have shown promising activity of dalbavancin against Gram-positive biofilms, including methicillin-resistant *S. aureus* (MRSA), methicillin-resistant *S. epidermidis* (MRSE), and vancomycin-susceptible enterococci. This review outlines the mechanisms regulating biofilm development in *Staphylococcus* and *Enterococcus* species and the clinical impact of biofilm-related infections. In addition, it discusses the clinical implications and potential therapeutic perspectives of the long-acting drug dalbavancin against biofilm-forming Gram-positive pathogens.

## Introduction

Gram-positive bacteria are the most common human pathogens associated with medical device-related infections and skin and soft tissue infections (SSTI) (Del Pozo and Patel, 2009; Kaye et al., 2019). *Staphylococcus aureus* is the leading pathogen of catheters and prosthetic related infections or in chronic ulcers, while *Enterococcus faecalis* and *Enterococcus faecium* have become prominent etiological agents of nosocomial infections worldwide (Tong et al., 2015; Guzman Prieto et al., 2016). The global prevalence of drug-resistant strains of Gram-positive bacteria is increasing. Specifically, community- and hospital-acquired infections caused by Methicillin-resistant *S. aureus* (MRSA) and vancomycin-resistant *E. faecium* (VRE) have become a serious concern (Huang et al., 2019).

The increasing prevalence of drug-resistant pathogens is further worsened by observing that pathogenic Gram-positive bacteria are particularly predisposed to form biofilms (Lebeaux et al., 2014). In hospital settings, biofilms are implicated in the pathogenesis of approximately 80% of chronic microbial infections or medical device-associated infections (MDI) (Römling and Balsalobre, 2012; Lebeaux et al., 2014). Furthermore, the biofilm lifestyle provides a broad and intrinsic multidrug tolerance allowing bacterial cells to survive a transient exposure to antibiotics without developing resistance (Di Domenico et al., 2019; Nguyen et al., 2020). Indeed, antibiotic resistance is genetically acquired by horizontal gene transfer or mutations, while the biofilm-related tolerance constitutes a multi-layered defense mechanism requiring slow-growing or non-dividing persister cells, poor antibiotic penetration, and adaptive stress responses (Lebeaux et al., 2014; Nguyen et al., 2020). Thus, the development of antibiotics with anti-biofilm activity represents a recognized need.

Dalbavancin is a semisynthetic lipoglycopeptide antibiotic structurally related to teicoplanin, with a long half-life and a unique dosage regimen, allowing for a single 1,500 mg dose or a two-dose (1,000 mg at day 1 and 500 mg at day 8) regimen (Boucher et al., 2014; Dunne et al., 2016). This antibiotic has a broad spectrum of action against Gram-positive cocci, including MRSA, and is approved for the treatment of SSTI (Arrieta-Loitegui et al., 2020). There is a growing amount of evidence that dalbavancin could be useful in other invasive Gram-positive infections that need prolonged intravenous treatments, including osteomyelitis, prosthetic joint infections (PJI), infective endocarditis, and catheter-related bacteremia (David et al., 2017). In addition, recent studies conducted *in vitro* and on human and animal models demonstrated that dalbavancin might also be effective against bacterial biofilms produced by staphylococci and enterococci (Knafl et al., 2017; Neudorfer et al., 2018; Silva et al., 2020; Žiemytė et al., 2020).

This review highlights the basic mechanisms regulating biofilm development in Gram-positive bacteria in the most clinically relevant infections along with the general principles of antimicrobial treatment of biofilm-associated infections. In addition, this review summarizes the current understanding and potential therapeutic activity of dalbavancin against *in vitro* and *in vivo* models of biofilm-related infections caused by Gram-positive bacteria.

### Role of Biofilms in Gram-Positive Infections

Immediately after placing a medical device into the patient’s body, biomaterials are rapidly coated with a conditioning film, a layer of the host organic elements absorbed onto the substratum. This conditioning layer generally provides a favorable substrate for bacteria to attach to the implant (Neoh et al., 2017). The adhesion of planktonic cells to the implant surface is the first step toward biofilm formation (Berne et al., 2018). In staphylococci, the adhesion to human tissue or indwelling medical devices is regulated by a large variety of surface-anchored proteins that bind to host tissues and cells, referred to as microbial surface components recognizing adhesive matrix molecules (MSCRAMMs) (Foster, 2019). These proteins are also present on *Enterococcus faecalis* and *Enterococcus faecium* and are required in the first step of human tissues colonization (Sava et al., 2010). There are two families of MSCRAMM molecules related to clumping factor A (ClfA) of *S. aureus* and serine-aspartate repeat protein G (SdrG) of *S. epidermidis* and another group referred to as the collagen-binding protein of *S. aureus* (the Cna family) (Foster, 2019). A single MSCRAMM protein performs several functions as binding to a diverse array of host ligands. Those molecules allow for unspecific attachment mediated by hydrophobic, electrostatic hydrogen-bonding, and van der Waals interactions with complementary receptors present in the conditioning film (Katsikogianni and Missirlis, 2004). In the early stages of biofilm growth, adherent cells are loosely associated with a surface. This is called the reversible attachment stage (Berne et al., 2018). During this phase complete eradication of the MDI can be achieved by the careful surgical debridement of tissue and bone marrow, and local antimicrobial therapy to eliminate planktonic bacteria (Høiby et al., 2015; Masters et al., 2019; da Silva et al., 2021). Indeed, in the early onset of a MDI, a major role is played by the potent virulence response of the infectious pathogen, which causes tissue destruction that may sometimes culminate in fulminant infections (Zimmerli et al., 2004; Masters et al., 2019; Seebach and Kubatzky, 2019). After the initial bacterial attachment to a surface, intracellular accumulation and biofilm maturation occur (Nguyen et al., 2020). The presence of a mature biofilm in either the local tissue or the implant requires more radical procedures (i.e., the complete removal of the implant) and prolonged therapies, often by the intravenous route, to remove the infection (Masters et al., 2019). During biofilm maturation, individual cells enter the irreversible attachment stage, and the bacterial cells produce the biofilm matrix. The irreversible attachment can be modulated by environmental factors such as pH, hydrodynamics, nutrient availability, temperature, osmolarity, oxygen, or other host factors (Palmer et al., 2007; Lister and Horswill, 2014; Berne et al., 2018). At this stage, mature biofilms are highly tolerant to host immune defenses, stresses, starvation, dehydration and cannot be eradicated by antibiotic treatments alone (Masters et al., 2019). In staphylococci and enterococci, similar factors contribute to the biofilm matrix composition including polysaccharides, proteins, teichoic and lipoteichoic acids, and extracellular DNA (eDNA) (Ch’ng et al., 2019; Karygianni et al., 2020). In staphylococci, the polysaccharide intercellular adhesin (PIA), also known as poly-N-acetyl glucosamine (PNAG), according to the chemical composition (Nguyen et al., 2020) is an important adhesive molecule during biofilm formation. The biosynthesis and accumulation of PIA on the bacterial surface are regulated by the intercellular adhesion (*ica*) gene locus products, including the *ica*A, *ica*D, *ica*B, and *ica*C genes. Although PIA plays a central role in staphylococcal biofilm, several studies have demonstrated that biofilm formation can be accomplished by *S. epidermidis* and *S. aureus* isolates in the absence of the *ica* operon by a PIA-independent mechanism (Otto, 2018; Nguyen et al., 2020). This process is particularly relevant to methicillin-resistant *S. aureus* (MRSA) strains where biofilm formation depends mainly on proteins rather than polysaccharides (McCarthy et al., 2015). In *E. faecalis*, the *dlt*ABCD operon is required to obtain d-alanine esters of lipoteichoic acids, which is an essential constituent of the Gram-positive bacterial cell wall (Ch’ng et al., 2019). A deletion mutant of the *dlt*A gene in *E. faecalis* produces significantly less biofilm *in vitro*, reduced adherence to epithelial cells, and increased susceptibility to cationic antimicrobial peptides. These results suggest a potential contribution of d-alanine of lipoteichoic acids in the pathogenesis of *E. faecalis* (Fabretti et al., 2006).

A mature biofilm is typically associated with chronic infections, which persist despite apparently adequate antibiotic treatment. Indeed, chronic infections may have a silent course for several months, perhaps years before the clinical symptoms appear. Chronic infections are mainly characterized by a local and persistent inflammatory response surrounding the biofilm. The infection’s signs and symptoms may vary depending on the organ’s function or implanted device (Høiby et al., 2015). An infection frequently characterized by less dramatic outcomes and persistent pain as the only manifestation (together with elevated C-reactive protein levels, although not always present) is a PJI (Masters et al., 2019). The majority of PJIs are thought to occur during surgery, due to the incidental introduction of skin commensals into the surgical site or onto the newly implanted device (Zimmerli et al., 2004). In a late PJI, biofilm cells remain quiescent and localized in the implant surface and the surrounding tissues. However, bacterial cells may continuously release from the biofilm by a dispersal process which may contribute to bacteremia and sepsis or disseminate to other implants within the body (Lister and Horswill, 2014). Dispersion of *S. aureus* from the biofilm into the environment is an active mechanism mediated by the production of extracellular enzymes or surfactants controlled by the activity of the accessory gene regulator (*agr*) system (Otto, 2018). As a quorum-sensing (QS) communication system, the *agr* locus regulates more than 70 genes in *S. aureus*, 23 of which are directly involved in its virulence (Otto, 2018). Specifically, the *agr* locus controls the expression of surface binding proteins, toxins, proteases, lipases, nucleases. The QS response also regulates matrix modification and dispersion in *E. faecalis* (Ch’ng et al., 2019). The Fsr (*Enterococcus faecalis* sensor regulator) QS system is a signal transduction system that controls the extracellular metalloprotease, gelatinase (GelE) (Ch’ng et al., 2019). Mutations in the *fsr* system or *gelE* revealed that gelatinase has an important role in *E. faecalis* biofilm formation and increased virulence in different animal infection models (Hancock and Perego, 2004; Sava et al., 2010). The released cell subpopulation, spreading from the original colony, is typically more virulent, with altered metabolic activity and antimicrobial susceptibilities than their biofilm and planktonic counterparts. Thus, the dispersed cell may result in more severe and persistent infections (Rumbaugh and Sauer, 2020).

### Clinical-Therapeutic Approach to Biofilm-Related Infections

#### General Principles

As a general concept, the clinical management of biofilm-related infections requires the complete removal of the infected device with surgical debridement followed by implant replacement along with targeted antibiotics against biofilms and planktonic cells (Høiby et al., 2015; Agarwal et al., 2020). Indeed, using antimicrobials with activity against biofilm positively influences the outcome, irrespective of the type of surgery performed (Gellert et al., 2020; Köder et al., 2020).

The purpose of surgery is to remove (i) the foreign body along with the surrounding patchy distributed biofilm, (ii) the infecting sessile germs, and (iii) the devitalized tissue, if any (Høiby et al., 2015; Izakovicova et al., 2019; Agarwal et al., 2020). At this stage, it is essential to put in place a targeted therapy (Table 1) to minimize the bacterial adhesion ability and prevent new biofilm formation by the residual microbial burden. One exception to the complete removal of a foreign body is the presence of early biofilm (i.e., implant infection within 3 weeks from implant placement or during concomitant bacteremia with further involvement of the implant). In this case, surgical debridement and the possibility of keeping the essential parts of the foreign body are recommended.

**TABLE 1:** Activity of different antibiotics against biofilm-growing Gram-positive bacteria.

Author, year	Antimicrobial agent	Penetration into the biofilm matrix	Activity against sessile cells
Landini et al. (2015) and Lázaro-Díez et al. (2016)	Beta-lactams	Reduced to a varying degree	None
Jo and Ahn (2016) and Di Domenico et al. (2019)	Quinolones	Yes	Active
Henry-Stanley et al. (2014) and Di Domenico et al. (2019)	Aminoglycosides	Reduced	Reduced
Darouiche et al. (1999)	Minocycline	Yes	Active
Tang et al. (2013) and Di Domenico et al. (2019)	Rifampicin	Yes	Active
Darouiche et al. (1994) and Doroshenko et al. (2014)	Vancomycin	Severely reduced	Not known
Leite et al. (2011) and Parra-Ruiz et al. (2012)	Daptomycin	Yes	Not known
Parra-Ruiz et al. (2012)	Linezolid	Yes	Reduced
Tang et al. (2012)	Fosfomycin	Yes	Active
Silva et al. (2020) and Žiemytė et al. (2020)	Dalbavancin	Reduced	Not known
Landini et al. (2015) and Lázaro-Díez et al. (2016)	Beta-lactams	Reduced to a varying degree	None
Jo and Ahn (2016) and Di Domenico et al. (2019)	Quinolones	Yes	Active
Henry-Stanley et al. (2014) and Di Domenico et al. (2019)	Aminoglycosides	Reduced	Reduced
Darouiche et al. (1999)	Minocycline	Yes	Active
Tang et al. (2013) and Di Domenico et al. (2019)	Rifampicin	Yes	Active
Darouiche et al. (1994) and Doroshenko et al. (2014)	Vancomycin	Severely reduced	Reduced
Leite et al. (2011) and Parra-Ruiz et al. (2012)	Daptomycin	Yes	Not known
Parra-Ruiz et al. (2012)	Linezolid	Yes	Reduced
Tang et al. (2012); Mihailescu et al. (2014), and Oliva et al. (2014)	Fosfomycin	Yes	Active
Silva et al. (2020) and Žiemytė et al. (2020)	Dalbavancin	Reduced	Not known

The so-called Debridement, Antibiotics, and Implant Retention (DAIR) approach is considered the optimal choice, showing a high clinical cure rate, especially in PJIs. During this procedure, radical debridement of all necrotic tissues, synovectomy, excision of sinus tracts and thorough irrigation with copious volumes of sterile saline is performed, combined with replacement of mobile, easily exchangeable prosthetic parts and targeted therapy for 6–12 weeks with the antibiotics listed in Table 1 (Høiby et al., 2015; Izakovicova et al., 2019; Agarwal et al., 2020). However, individual patients may not be candidates for any device removal, i.e., subjects with an unacceptably high risk for mortality due to surgery, subjects with a limited life expectancy, or those refusing device explanation (Peacock et al., 2018).

In these cases, alternative options such as conservative management with device retention and long-term suppressive antimicrobial therapy may be considered (Segreti et al., 1998; Pavoni et al., 2002, 2004; Peacock et al., 2018; Izakovicova et al., 2019).

#### Antimicrobial Treatment

Biofilm-embedded bacteria are up to 100–1,000 times less susceptible to antibiotics than their planktonic counterpart (Sharma et al., 2019). In this context, conventional *in vitro* susceptibility testing methods are unsuitable; therefore, when choosing antimicrobials, the key step is to consider their anti-biofilm activity. Table 1 attempts to list the characteristics of the most common antibiotics with efficacy against biofilm and summarizes the current knowledge on the two main steps of antimicrobial activity in this setting, i.e., diffusion through the biofilm matrix and activity against sessile bacterial cells (Ciofu et al., 2017; Izakovicova et al., 2019; Abad et al., 2020). There is also a third, preliminary stage, which has been described for rifampicin, minocycline, linezolid, macrolides, colistin, and dalbavancin: the ability to prevent/impair bacterial adhesion on inert surfaces and subsequent biofilm deposition (Parra-Ruiz et al., 2012; Ciofu et al., 2017; Albano et al., 2019; Izakovicova et al., 2019; Di Pilato et al., 2020). The anti-staphylococcal biofilm agent *par excellence* is rifampicin, followed by two drugs with similar potentials, the long-acting agents rifabutin and rifapentine. Nevertheless, rifampicin should not be used as monotherapy due to the risk of rapid development of *in vivo* resistance (Høiby et al., 2015; Ciofu et al., 2017; Izakovicova et al., 2019). Therefore, the association with other anti-staphylococcal drugs is recommended not only on account of their frequent synergistic activity but also to minimize the development of resistance. To this end, since the emergence of rifampicin resistance is proportional to the bacterial burden, there is also a recommendation to initiate the antimicrobial therapy with partner antibiotics such as oxacillin, daptomycin, or dalbavancin, to reduce the bacterial load, and then after 3–5 days add rifampicin in association (Høiby et al., 2015; Izakovicova et al., 2019). In *in vitro* and animal models, rifampicin showed activity also against biofilm formed by *Cutibacterium acnes* (Furustrand Tafin et al., 2012). However, in a recent study on PJI caused by *C. acnes*, rifampicin therapy did not seem to improve outcomes, suggesting the need for additional studies on humans (Vilchez et al., 2021).

The choice of antibiotics against enterococcal biofilms is limited, and, with this regard, no activity of rifampicin has been shown (Oliva et al., 2014). On the other hand, the “old” antibiotic fosfomycin, which has been recently rediscovered mostly to treat multidrug-resistant bacteria, showed high activity against both staphylococcal and enterocococcal biofilms (Mihailescu et al., 2014; Oliva et al., 2014; Zheng et al., 2019). Nevertheless, similarly to rifampicin, fosfomycin monotherapy allows the rapid selection of resistant variants, and therefore it should always be used in combination. As an alternative to the traditional approach combining surgery plus antibiotics, chronic suppressive therapy is selected when a patient’s general conditions or the technical difficulties connected with the surgery are such as to preclude both removal and replacement of the foreign body and debridement. After initial targeted therapy with antibiotics by the intravenous route, the subsequent step is to keep the infection “silent” by administering a prolonged oral therapy for an indefinite duration, including, amongst others, the use of minocycline and/or trimethoprim/sulfamethoxazole and/or fluoroquinolones, according to the causative agent and the patient’s characteristics (Pavoni et al., 2004; Izakovicova et al., 2019; Qu et al., 2019). This approach is mainly described in orthopedic implant infections and more rarely in the management of cardiac implant or vascular graft infections (Segreti et al., 1998; Pavoni et al., 2002, 2004; Peacock et al., 2018; Izakovicova et al., 2019; Blomström-Lundqvist et al., 2020). However, some patients do not even tolerate this approach, mainly due to side effects during suppressive therapy (Segreti et al., 1998; Spaziante et al., 2019). In such cases, an alternative option, currently under study, is the use of dalbavancin for a prolonged period with intravenous administrations at intervals of up to 12 weeks apart, driven by the determination of the serum bactericidal assay, as recently described (Spaziante et al., 2019). Another alternative strategy aimed to retain in place the foreign body is represented by intra-lock therapy, which refers to the treatment of catheter-related bloodstream infections and may be considered only if there are absolute contraindications to catheter removal. Antibiotics for the lock therapy are at high concentrations (up to 100–1,000 times higher than the MIC). They may include aminoglycosides, beta-lactams, fluoroquinolones, glycopeptides, daptomycin, linezolid, minocycline, and tigecycline, with the largest evidence for gentamicin and vancomycin (Justo and Bookstaver, 2014). However, when selecting antimicrobials for the intra-lock therapy, additional characteristics such as stability, compatibility with anticoagulants, selection of resistance, rate of systemic absorption with possible consequent side effects, and cost-effectiveness should be considered (Justo and Bookstaver, 2014).

Overall, foreign body infections are associated with increasingly complex implications as the number and variety of implantable devices grow, especially in subjects with critical conditions or peculiar psychological profiles (i.e., refusal of surgery). Therefore, clinicians are often called upon to “invent” new therapeutic strategies (Pavoni et al., 2002).

It should also be taken into account that the traditional antimicrobial strategy toward implant-associated infections requires prolonged antimicrobial administration, largely by intravenous route. Consequently, multiple or prolonged hospitalizations may be required, therefore exposing patients, especially those with several comorbidities or immune suppression, to the risk of acquiring nosocomial and multidrug-resistant microorganisms (Buke et al., 2007). This is especially true if elderly patients are considered and if additional invasive procedures are necessary (i.e., central venous access placement for antibiotic administration). Therefore, alternative strategies such as the Outpatient Parenteral Antibiotic Therapy (OPAT) and the long-acting dalbavancin are increasingly adopted to overcome these drawbacks.

### Potential Role of Dalbavancin in the Treatment of Gram-Positive Biofilms

Dalbavancin currently represents the ultralong acting agent with the most extensive experience of use (Baldoni et al., 2013). Specifically, dalbavancin is a novel semisynthetic antimicrobial agent belonging to the second-generation lipoglycopeptides family approved by the FDA and the EMA for the treatment of acute bacterial skin and skin structure infections (ABSSSI). Dalbavancin inhibits the late stages of peptidoglycan synthesis, interrupting bacterial cell wall synthesis by binding to the terminal D-alanyl–D-alanine terminus of pentapeptide peptidoglycan precursors, resulting in bactericidal activity against most Gram-positive microorganisms. Notably, recent data showed that dalbavancin MIC_90_ values remained unchanged, being ≤ 0.06 μg/mL against different species of Gram-positive germs, with overall low values: *Staphylococcus aureus* (MIC50/90 0.03 mg/L for both MSSA and MRSA), vancomycin-susceptible *Enterococcus faecalis* (MIC50/90, 0.03/0.06 mg/L), β-hemolytic streptococci (MIC50/90, 0.008/0.015 mg/L), and *Streptococcus anginosus* group (MIC50/90, ≤ 0.004/ ≤ 0.004 mg/L) (Jones et al., 2013). Interestingly, dalbavancin resistance during or immediately after the antibiotic treatment was evidenced in only two cases (Kussmann et al., 2018; Werth et al., 2018), with the hypothesized mechanisms underlying the reduced susceptibility being linked to an increase in cell-wall thickness (Kussmann et al., 2018). From a clinical point of view, dalbavancin has been approved for use as a 2-dose regimen (1,000 mg IV on day 1 and 500 mg IV on day 8) or 1-dose regimen (1,500 mg IV) for the treatment of ABSSSI, with an efficacy equal to and fewer adverse reactions than the standard treatment with vancomycin followed by oral linezolid (Soriano et al., 2020). Besides being a good choice in managing SSTI, dalbavancin may represent a valuable option for other invasive Gram-positive infections requiring prolonged intravenous treatments, including osteomyelitis, PJI, infective endocarditis, and catheter-related bacteremia (David et al., 2017). Retrospective analyses of patients with these types of infections being treated with dalbavancin showed a favorable outcome in most cases and an excellent safety profile (Tables 2, 3). The efficacy of dalbavancin was also proved in vulnerable patients with osteomyelitis or non-complicated bacteremia where the first-line antimicrobial therapy failed (Bork et al., 2019). Subsequent successful use of dalbavancin in patients with infective endocarditis was also reported (Tobudic et al., 2018). Since endocarditis or MDIs are at high risk of biofilm development, it has been speculated that dalbavancin efficacy in such patients may be linked to a direct mechanism on biofilm eradication. Different studies evaluated the *in vitro* activity of dalbavancin against biofilms formed by Gram-positive infections. In *in vitro* biofilm models of Gram-positive cocci, dalbavancin inhibited biofilm formation at low concentrations in a broad number of *S. aureus*, *S. epidermidis*, and enterococci (Fernández et al., 2016; Neudorfer et al., 2018). These values were lower than those observed for other agents such as vancomycin and daptomycin. An exception was represented by vancomycin-resistant strains, which showed very high minimum biofilm bactericidal concentrations for all tested agents, including dalbavancin (Fernández et al., 2016; Neudorfer et al., 2018). A recent study evaluated the time-kill kinetics of dalbavancin against biofilm formed by *S. aureus* and coagulase-negative staphylococci (CoNS) (Di Pilato et al., 2020). Dalbavancin and vancomycin, used at a concentration achievable *in vivo* in bone tissue (1, 4, and 16 μg/mL) for 7 days, showed concentration and time-dependent activities against all tested strains. Besides, dalbavancin showed a greater reduction of biofilm-embedded bacteria in most strains studied, especially at 4 μg/mL and 16 μg/mL. In biofilms formed on titanium and cobalt chrome disks, dalbavancin was more active than vancomycin at medium concentrations (4 μg/mL), which are easily reached in bone tissue (Dunne et al., 2015). The activity of dalbavancin against *S. aureus* and *S. epidermidis* biofilms was compared to other antimicrobials (linezolid, rifampicin, vancomycin, cloxacillin). Notably, the minimal biofilm inhibitory concentration (MBIC) of dalbavancin ranged from 0.5 to 2 μg/mL, and in combination with rifampicin, showed the highest biofilm inhibitory effect. In addition, dalbavancin was able to eradicate 6–9-h old biofilms at concentrations of 8–32 μg/mL. The other antimicrobials showed no activity against biofilms formed by *S. aureus*. Dalbavancin was effective against *S. epidermidis* biofilm; however, cloxacillin plus rifampicin showed lower MBIC values (Žiemytė et al., 2020). A recent study analyzing the effect of different antibiotics on biofilm-producing MRSA strains from patients with SSTI showed that dalbavancin was 16 and 8 times more active than linezolid and vancomycin, respectively, with an MBIC_90_ of 0.5 μg/mL (range 0.12–0.5 mg/L) (Sivori et al., 2021).

**TABLE 2:** Dalbavancin in the treatment of bone and joint infections: an analysis of the literature.

Author, year	Study design, cases	Dose° and duration of dalbavancin therapy	Clinical success	Adverse events
Almangour et al. (2017)	Case report, spondylodiscitis caused by MRSA	1 × 1,000 mg loading dose, then 500 mg/week × 6 times	Yes, at the end of therapy (follow up: no)	None
Bouza et al. (2018)	Retrospective: 12 OM, 5 PJI	Varying: mean of 4 doses (range 1–9), 3-week duration (range 1–24)	92% for OM, 80% for PJI (follow up: 1 month)	13%, mild
Rappo et al. (2018)	Prospective randomized; Dal: 70 vs. ST: 10; 80 OM	1 gr IV on days 1 and 8	96% dal vs. 88% ST	None for dal
Tobudic et al. (2018)	Retrospective: 20 OM, 8 PJI; 14 SPD, 4 SAR	Varying: median duration of 8 weeks (range 4–32)	60% for OM, 38% for PJI; 50% for SPD; 100% for SAR (follow up: 6 months)	2.8%, mild
Wunsch et al. (2019)	Retrospective: 30 OM, 32 PJI	Varying: mean of 3 doses (range 1–32)	89% for OM, 91% for PJI (at the end of therapy, no follow up)	3%, mild
Bryson-Cahn et al. (2019)	Retrospective: 7 OM	Varying: median of 1 dose (range 1–5)	71% (follow up: 1 year)	None
Morata et al. (2019)	Retrospective: 19 OM/SPD, 26 PJI	Varying: median of 5 doses (IQR 3–8)	90% for OM/SPD; 65% for PJI (follow up: 6 months)	11%, mild
Almangour et al. (2019)	Retrospective: 29 OM/SPD, 2 PJI	Varying: median of 3 doses (range 1–14)	93% for OM/SPD, 100% for PJI (follow up: 3 months)	None
Bartoletti et al. (2019)	Retrospective: 15 sternal OM post-cardiac surgery	Varying: median of 4 doses	93% (follow up: 6 months) (follow up: 1 month)	None
Bork et al. (2019)	Retrospective: 11 OM, 1 PJI	Varying: median of 3 doses (IQR 4.5)	55% for OM; 100% for IPA	13%, mild
Streifel et al. (2019)	Retrospective: 11 OM	Varying: mean of 2.7 weeks	76% (follow up: 1 month)	8%, mild
Almangour and Alhifany (2020)	Retrospective case-control Dal: 11 OM vs. ST 11 OM	Varying: mean of 2 doses	100% in both arms	None
Loupa et al. (2020)	Case report, diabetic foot OM caused by multi-drug resistant *Enterococcus faecium*	2 × 1,500 mg in combination with oral lin and intravenous tig	Yes, at the end of the therapy	None
Veve et al. (2020)	Retrospective: osteoarticular infection (OM, PJI, septic arthritis), infective endocarditis or other bloodstream infection 70 dal and 145 ST	Varying: the most frequent (34%) was 1,500 mg for two doses 1 week apart	Lower rate of 90-day infection-related readmission in dal treated (17%) vs. ST (28%)	3% dal vs. 14% ST
Matt et al. (2021)	Retrospective: 17 PJI	Varying: the most frequent (8 patients) was 1,500 mg at Day 1 and 1,500 mg at Day 7	47% after a median follow-up of 299 days	Not specified
Navarro-Jiménez et al. (2021)	Retrospective, descriptive study: 23 OM (diabetic foot infection)	Varying: the most frequent (8 patients) was 1,000 mg followed by 500 mg weekly for 5 weeks	87% at 90 days after completion of dal	13%, mild

*Clinical success is defined as the disappearance of any clinical, laboratory, and microbiological evidence of persistent or relapsing infection at the last clinical assessment after dalbavancin discontinuation;° intravenous administration; OM, osteomyelitis; PJI, prosthetic joint infection; SPD, spondylodiscitis; SAR, septic arthritis; lin, linezolid; dal, dalbavancin; tig, tigecycline; ST, standard therapy.*

**TABLE 3:** Dalbavancin in the treatment of infective endocarditis: an analysis of the literature.

Author, year	Study design, cases	Prior therapy	Dose° and duration of dalbavancin therapy	Clinical response*	Adverse events
Steele et al. (2018)	1 NVE-rt in pregnancy, MRSA	Van 5 dd, dap 27 dd	1 × 1,000 mg loading dose, then 500 mg/week × 3 times	Failure; success with cef + dap	The emergence of van-intermediate/tel non-susceptible MRSA during therapy with dal
Tobudic et al. (2018)	Retrospective: 27 cases of endocarditis: 59% NVE, 22% PVE, 19% PME; surgical therapy: 80% in PVE/PME.	89% prior therapies, with dal initiated after bacteremia clearance	33%: 1,000 mg load, then 500 mg/week; 66% 1,500 mg load, then 1,000 mg/week. Mean duration: 42 dd	92% clinical success	1 nausea; 1 increased serum creatinine
Kussmann et al. (2018)	1 PME, MSSA (PM not removable?)	5 prior therapies for over 1 year	Doses and dosing intervals not specified; duration of about 30 weeks	Not reported; isolation of strains not sensitive to dal from blood culture and explanted PM	Resistance to dal, case of PM not explanted
Dinh et al. (2019)	Retrospective: 9 NVE, 10 PVE	99% prior therapies with a median duration (IQR) of 23 dd; dal initiated after 2.5 lines of therapy (mean)	53%: 1 or 2 doses of 1,500 mg weekly	73% clinical success	Stop dal: = 0%; 2 hypersensitivity reactions°°
Morrisette et al. (2019)	Retrospective: 5 NVE	91% prior therapies with a mean duration of 27 dd; 30% combo∧	60% dal 1 × 1,500 mg dose at end of therapy	100% clinical success	Infusion reactions, phlebitis at the infusion site
Bryson-Cahn et al. (2019)	Retrospective: 9 NVE-rt, 0MSSA, 7 MRSA	100% prior therapies; bacteremia clearance before initiating dal	6 cases of 1 × 1,000 mg dose; 3 cases of two doses (1,000 mg then 500 mg after 7 dd)	5/9 clinical success; 4/9 discharged patients improved but were lost at follow-up	None
Wunsch et al. (2019)	Retrospective: 15 NVE, 6 PVE, 4 PME; 3 cases of associated spondylodiscitis	100 prior therapies; 60% combo	9 cases of 1 × 1,500 mg dose; 8 cases of multiple weekly doses of 500 mg, preceded by a loading dose of 1,000 mg	89% clinical success; 1 death during therapy	1 hypertension during infusion; 1 muscle weakness; 1 vertigo
Hidalgo-Tenorio et al., 2019	Retrospective: 83 (59.04% bloodstream infection, 49.04% infective endocarditis (44.04% PVE, 32.4% NVE, 23.5% pacemaker lead)	Dap (68.6%), cex (28.6%), van (22.9%), lin (8.6%)	Varying: the most frequent (12 patients) was 1,500 mg (1 day)	In hospital clinical cure in all patients; at 12 months, 2.9% therapeutic failure	4.8%, mild
Spaziante et al. (2019)	1 PVE MRSE + *S. mitis*, considered to be inoperable	Pip/taz + dap, then cef + dap. Dal initiated after bacteremia clearance	1,500 mg on days 1, 7, 42, 112, 189, 255″, 315″, 370″ (the frequency of infusions was guided by *SBP*-values ≤ 1:8)	Net clinical and PET/CT improvement, no relapse after more than 1 year from dal discontinuation^§^	None
Veve et al. (2020)	Retrospective: osteoarticular infection (OM, PJI, septic arthritis), infective endocarditis or other bloodstream infection 70 dal and 145 ST	Not specified for the infective endocarditis group (all patients in the dal group received prior antibiotics during the hospitalization preceding definite dal therapy)	Varying: the most frequent (34%) was 1,500 mg for two doses 1 week apart	Lower rate of 90-day infection-related readmission in dalbavancin treated (17%) vs. ST (28%)	3% dal vs. 14% ST

*Clinical success is defined as the disappearance of any clinical, laboratory and microbiological evidence of persistent or relapsing infection at the last clinical assessment after dalbavancin discontinuation;° intravenous administration; NVE, native valve endocarditis; NVE-rt, right-sided native valve endocarditis (unless specified, NVEs should be considered to be left-sided); PVE, prosthetic valve endocarditis; dd, days; cef, ceftaroline; cex, ceftriaxone; dal, dalbavancin; dap, daptomycin; lin, linezolid; tel, telavancin; van, vancomycin; stop dal, discontinuation of dalbavancin due to an adverse event; ° °fever, rash, chills and/or fever during the first infusion; ∧ = dalbavancin in combination with other antibiotic(s); PME, pacemaker endocarditis; MSSA, methicillin-sensitive S. aureus; MRSA, methicillin-resistant S. aureus; MRSE, methicillin-resistant S. epidermidis; S. mitis, Streptococcus mitis.”, infusions performed after the article was written; SBP, serum bactericidal power, when the value was ≤ 1:8 dalbavancin infusion was performed. ^§^ Reported in the article: note of the author (MV) who followed up the case.

A few *in vivo* experimental models evaluated dalbavancin activity in biofilm prevention and treatment. Darouiche and Mansouri evaluated biofilm prevention in a rabbit model inoculated with *S. aureus* and treated with either dalbavancin or vancomycin (Darouiche and Mansouri, 2005). The percentage of colonized devices was comparable in the vancomycin and control group (47%). In contrast, the dalbavancin group showed a lower trend in device colonization (28%), although not statistically significant (Darouiche and Mansouri, 2005). Nevertheless, the rate of foreign body contamination in rabbits receiving placebo was around 50% (lower than other animal models), thus questioning the validity of the model and its discriminatory power for assessing the efficacy of antimicrobials. In 2013, Baldoni et al. tested dalbavancin activity, alone and in combination with rifampicin, on MRSA biofilm in an animal model of tissue-cage infection. Dalbavancin did not show antagonist or synergistic activity with rifampicin; however, when used in combination with rifampicin, it was able to eradicate the biofilm and achieve cure rates of 25–36% compared to monotherapy. In addition, dalbavancin prevented the insurgence of rifampicin resistance (Baldoni et al., 2013). A more recent paper by Silva et al. (2020) evaluated dalbavancin activity in a rat model of implant-associated orthopedic infection by MRSA. Efficacy was assessed at 7 and 14 days after dalbavancin administration, showing a significant reduction in bacterial colonies on bone tissue and implant.

However, some limitations from these pre-clinical reports should be highlighted: the dosages of dalbavancin in some of the *in vivo* models may have provided lower antibiotic exposure compared to human pharmacokinetics; the chosen animal models may not represent the ideal *in vivo* condition for biofilm growth; more data on the combination therapies, especially with rifampicin, would be essential to fully understand the possible role of dalbavancin in hard-to-treat infections.

Furthermore, the clinical evidence available so far derives mostly from retrospective observational studies or case reports (Tables 2, 3). The only prospective randomized study comparing dalbavancin with the standard of care included patients with osteomyelitis, a condition that is associated with biofilm but that does not involve a foreign body (Rappo et al., 2018). Therefore, based on these promising but still preliminary data, additional prospective randomized trials evaluating the role of dalbavancin in patients with implant-associated infections are strongly encouraged.

## Conclusion

With an aging population and the resulting increase in diseases such as cancer and diabetes, together with the development of new implantable medical devices, there is an increasingly growing incidence of chronic infections typically associated with biofilm formation.

The current management of several biofilm-related Gram-positive infections requires prolonged antibiotic therapy and, in the majority of the cases, the complete removal of the implant.

Amongst the available antimicrobials with different degrees of activity against biofilm, dalbavancin seems to provide effective therapy in a significant proportion of cases due to its ultra-long activity and effectiveness in the setting of MDIs with a relatively low number of adverse effects. Furthermore, its ease of administration allows to accelerate patients’ discharge from hospital and increase patients’ compliance to the therapy, thus reducing both healthcare costs and the risks of developing multidrug-resistant bacterial infections due to prolonged hospital stay.

## Author Contributions

All authors designed the study, wrote the manuscript, interpreted the clinical data, contributed to the article, and approved the submitted version.

## Conflict of Interest

The authors declare that the research was conducted in the absence of any commercial or financial relationships that could be construed as a potential conflict of interest.

## Publisher’s Note

All claims expressed in this article are solely those of the authors and do not necessarily represent those of their affiliated organizations, or those of the publisher, the editors and the reviewers. Any product that may be evaluated in this article, or claim that may be made by its manufacturer, is not guaranteed or endorsed by the publisher.
